# Measuring Human Corneal Stromal Biomechanical Properties Using Tensile Testing Combined With Optical Coherence Tomography

**DOI:** 10.3389/fbioe.2022.882392

**Published:** 2022-05-20

**Authors:** Yi Song, Di Wu, Min Shen, Like Wang, Congzheng Wang, Yong Cai, Chao Xue, George P.M. Cheng, Yongping Zheng, Yan Wang

**Affiliations:** ^1^ Clinical College of Ophthalmology, Tianjin Medical University, Tianjin, China; ^2^ Tianjin Eye Hospital, Tianjin Key Lab of Ophthalmology and Visual Science, Tianjin Eye Institute, Tianjin, China; ^3^ Pacific University College of Optometry, Forest Grove, OR, United States; ^4^ School of Mechanical Engineering, Tianjin University, Tianjin, China; ^5^ Department of Biomedical Engineering, The Hong Kong Polytechnic University, Hong Kong, China; ^6^ School of Optometry, The Hong Kong Polytechnic University, Hong Kong, China; ^7^ Research Institute for Smart Ageing, The Hong Kong Polytechnic University, Hong Kong, China; ^8^ Nankai University Eye Institute, Nankai University Affiliated Eye Hospital, Nankai University, Tianjin, China

**Keywords:** cornea, biomechanical properties, tensile testing, optical coherence tomography, dynamic response parameters, myopia

## Abstract

**Purpose:** To investigate the *ex vivo* elastic modulus of human corneal stroma using tensile testing with optical coherence tomography (OCT) imaging and its correlation with *in vivo* measurements using corneal visualization Scheimpflug technology.

**Methods:** Twenty-four corneal specimens extracted from stromal lenticules through small incision lenticule extraction were cut into strips for uniaxial tensile tests. *In vivo* corneal biomechanical responses were evaluated preoperatively using the corneal visualization Scheimpflug technology (CorVis ST). The correlation of the elastic modulus with clinical characteristics and dynamic corneal response parameters were analyzed using *Spearman*’*s* correlation analysis.

**Results:** The mean low strain tangent modulus (LSTM) of the human corneal stroma was 0.204 ± 0.189 (range 0.010–0.641) MPa, and high strain tangent modulus (HSTM) 5.114 ± 1.958 (range 2.755–9.976) MPa. Both LSTM (*r* = 0.447, *p* = 0.029) and HSTM (*r* = 0.557, *p* = 0.005) were positively correlated with the stress-strain index (SSI). LSTM was also positively correlated with the A1 deflection length (*r* = 0.427, *p* = 0.037) and A1 deflection area (*r* = 0.441, *p* = 0.031). HSTM was positively correlated with spherical equivalent (*r* = 0.425, *p* = 0.038).

**Conclusions:** The correlation of corneal elastic modulus with A1 deflection parameters and SSI may indicate a relationship between these parameters and tissue elasticity. The HSTM decreased with the degree of myopia. Combining tensile test with OCT may be a promising approach to assess corneal biomechanical properties.

## Introduction

As an important component of the ocular wall, the cornea provides almost 2/3 ocular refractive power and a protection of inner ocular tissues, and helps maintain the physiological shape of the eye. It is also a biological soft tissue with complex biomechanical properties such as nonlinear elasticity, viscoelasticity, anisotropy, and heterogeneity. Corneal biomechanical properties play a vital role in its shape and function, and are generally used to interpret corneal physiological phenomena, diseases and its responses to treatments (e.g., refractive surgeries) (Ruberti et al., 2011; Kling and Hafezi, 2017; Ma et al., 2018; Blackburn et al., 2019). Thus, there is a demand for an accurate and reliable method for assessing the mechanical characteristics of the cornea.

Currently, there are two available devices in clinical practice to characterize *in vivo* corneal biomechanics - the ocular response analyzer (ORA; Reichert Ophthalmic Instruments, Buffalo, United States) and the Corneal visualization Scheimpflug technology (CorVis ST; OCULUS Optikgeräte GmbH; Wetzlar, Germany). The CorVis ST detects the corneal deformation imaging during a Gaussian-distributed air impulse by a high-speed Scheimpflug camera. Various corneal deformation parameters are obtained, as well as material stiffness parameters such as SP A1 and SP HC. More recently, the stress-strain index (SSI) has been developed and validated to estimate corneal biomechanical behavior (Eliasy et al., 2019). Nevertheless, these metrics cannot directly reflect intrinsic mechanical properties, such as the elastic modulus, and most of them relate to intraocular pressure (IOP) and corneal pachymetry (Vinciguerra et al., 2016), which may confuse clinicians.

Laboratory corneal biomechanical evaluations include destructive methods, such as tensile test, inflation test, and atomic force microscopy as well as non-destructive techniques with potential clinical applicability, such as electronic speckle pattern interferometry, ultrasonic elastography, and Brillouin microscopy. Uniaxial tensile testing is a straightforward assessment of corneal mechanical properties *in vitro* by applying a load to a sample and measuring its relative deformation (Elsheikh and Alhasso, 2009). Commonly used samples are corneal strips with specific length and width. In order to calculate the applied stress, the force (load) needs to be divided by the area resisting the force; thus, the measurement of the sample thickness becomes the main challenge (Robinson and Durand-Smet, 2020). Previous studies have used ultrasound pachymetry (Elsheikh et al., 2008; Bell et al., 2018) or surgical parameters (Xue et al., 2018) to obtaining the sample thickness. However, these approaches have some limitations in terms of precision due to tissue swelling during sample preservation, preparation, and the experimental process.

Optical coherence tomography (OCT) is a key ophthalmologic imaging technique can provide rapid, noninvasive, high-resolution *in vivo* imaging of corneal structures, which enables its broad diagnostic and therapeutic applications (Ang et al., 2018; Spaide et al., 2018; Wang et al., 2019). In addition, OCT can assist tissue biomechanical detection when combined with an air-puff (Huang et al., 2011) or shear wave elastography (Wang and Larin, 2015). Among all categories of OCT devices, spectral-domain OCT (SD-OCT) has the advantages of high-speed acquisition and high axial resolution. Recently, Wang et al.(Wang et al., 2018) reported the use of SD-OCT in an inflation testing system for the assessment of corneal mechanical properties.

Given its advantage of real-time and high-resolution imaging, OCT can also be applied to tensile testing to obtain the exact corneal sample thickness. To date, no study has integrated these two testing methods. Hence, the purpose of this study was to investigate the *ex vivo* elastic modulus of the human corneal stroma by uniaxial tensile testing combined with OCT imaging and to determine its correlation with *in vivo* CorVis ST parameters.

## Materials and Methods

### Preparation of Specimens

Specimens were human corneal strips obtained from small incision lenticule extraction (SMILE) surgery. The study was approved by the Ethics Committee of Tianjin Eye Hospital and was carried out in accordance with the Declaration of Helsinki. Prior to this study, all participants signed the informed consent to agree with use of clinical data. A comprehensive ophthalmic examination including slit-lamp microscopy, non-contact tonometry and anterior segment tomography was conducted preoperatively to confirm a healthy cornea. The exclusion criteria were as follows: 1) keratoconus or suspected keratoconus, 2) active ocular or systemic diseases, 3) previous ocular trauma or surgeries, and any other condition that could affect the health of the cornea.

Twenty-four corneal specimens from 22 patients (7 men; 15 women) with a mean age of 23.96 ± 5.27 (range 17–36) years were included in the study. The mean preoperative sphere was −4.96 ± 1.29 D, and the mean astigmatism −0.38 ± 0.18 D. All the astigmatism was with the rule and less than −1.00 D to ensure a regular geometric configuration of the corneal strip. Demographic and clinical data are summarized in Table 1.

**TABLE 1:** Demographic and clinical characteristics (*n* = 24).

Parameters	Mean ± SD (Range)
**Age (y)**	23.96 ± 5.27 (17–36)
**Sphere (D)**	−4.96 ± 1.29 (-8.00 to −2.75)
**Cylinder (D)**	−0.38 ± 0.18 (−0.75to 0)
**SE (D)**	−5.15 ± 1.28 (−8.125–−2.875)
**Km (D)**	42.76 ± 1.41 (40.13–45.55)
**CCT (μm)**	543.96 ± 21.65 (517–595)
**IOP (mmHg)**	16.19 ± 2.25 (12.3–21.3)

SD, standard deviation; SE, spherical equivalent; Km, mean keratometry; CCT, central corneal thickness; IOP, intraocular pressure.

The SMILE procedures were performed by the same experienced physician at Tianjin Eye Hospital using a VisuMax femtosecond laser system (Carl Zeiss Meditec AG, Jena, Germany). A corneal stromal lenticule was created by femtosecond laser, and then dissected and extracted through a small incision by the surgeon. The lenticule was then preserved in corneal storage medium (Eusol-C; Alchima, Padova, Italy) at a temperature of 4°C before preparation for the experiment. All specimens were tested in <2 h to avoid tissue swelling.

Prior to testing, a 1 mm-width strip was cut from the central region of the corneal lenticule with a customized double-blade knife. The strip length varied slightly with the diameter of the lenticule and was approximately 6.6 mm.

### CorVis Scheimpflug Technology Measurement

All patients underwent *in vivo* biomechanical examination preoperatively using CorVis ST (*ver. 1.6r2187*). Measurement quality was checked by the reading in the QS window, and the data with an “OK” reading were considered usable.

**FIGURE1:**
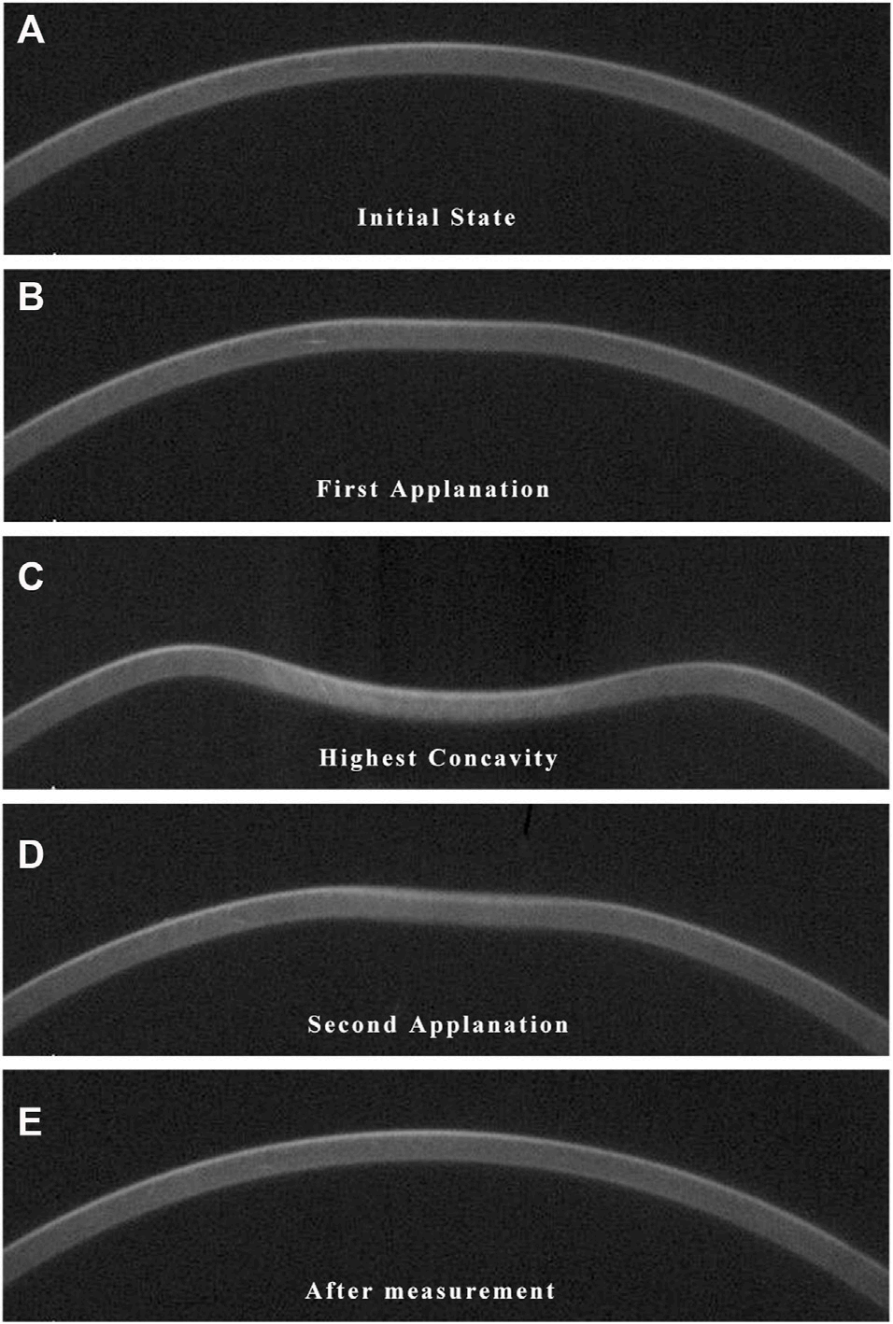
Images of corneal deformation during CorVis ST measurement. When applied by an air pulse, the cornea inwards into a concavity state and finally returns to its original shape through the following phases. **(A)** The initial convex state. **(B)** The first applanation. **(C)** The highest concavity. **(D)** The second applanation. **(E)** The final convex state.

The details and principles of CorVis ST measurement have been described elsewhere (Roberts, 2014; Wang et al., 2016). Briefly, the CorVis ST captures the corneal dynamic deformation applied by an air-puff force. As illustrated in Figure 1, the entire deformation process starts with the ingoing phase, in which the cornea passes from a convex state through the first applanation into a convave state, and finally reaches the point of highest concavity. Then the cornea achieves the oscillation phase, after which it returns to its initial shape through the second applanation. Three important events during the deformation process are the moment of the first applanation, the highest concavity and the second applanation. Corneal dynamic response parameters describing these three events were acquired, including the deformation amplitude, deflection length and deflection amplitude at the first applanation (A1 Deformation Amp., A1 Deflection Length, A1 Deflection Amp.), second applanation (A2 Deformation Amp., A2 Deflection Length, A2 Deflection Amp.) and highest concavity (HC Deformation Amp., HC Deflection Length, HC Deflection Amp.), corneal velocity at the first (A1 velocity) and second applanation (A2 velocity), time from the initiation of air puff until the first applanation (A1 Time), second applanation (A2 Time) and maximum deformation (HC Time), whole eye movement (WEM), peak distance and radius of curvature (Radius), the maximal value of the ratio between deformation amplitude at the apex and that at 2 mm (DA Ratio 2 mm) from the corneal apex*,* Ambrósio relational thickness to the horizontal profile (ARTh), integrated radius. Corneal stiffness parameters calculated based on the dynamic response parameters were also obtained, including stiffness parameter at first applanation (SPA1) and highest concavity (SP HC)*,* and stress-strain index (SSI).

### Uniaxial Tensile Testing With Optical Coherence Tomography Imaging

**FIGURE 2:**
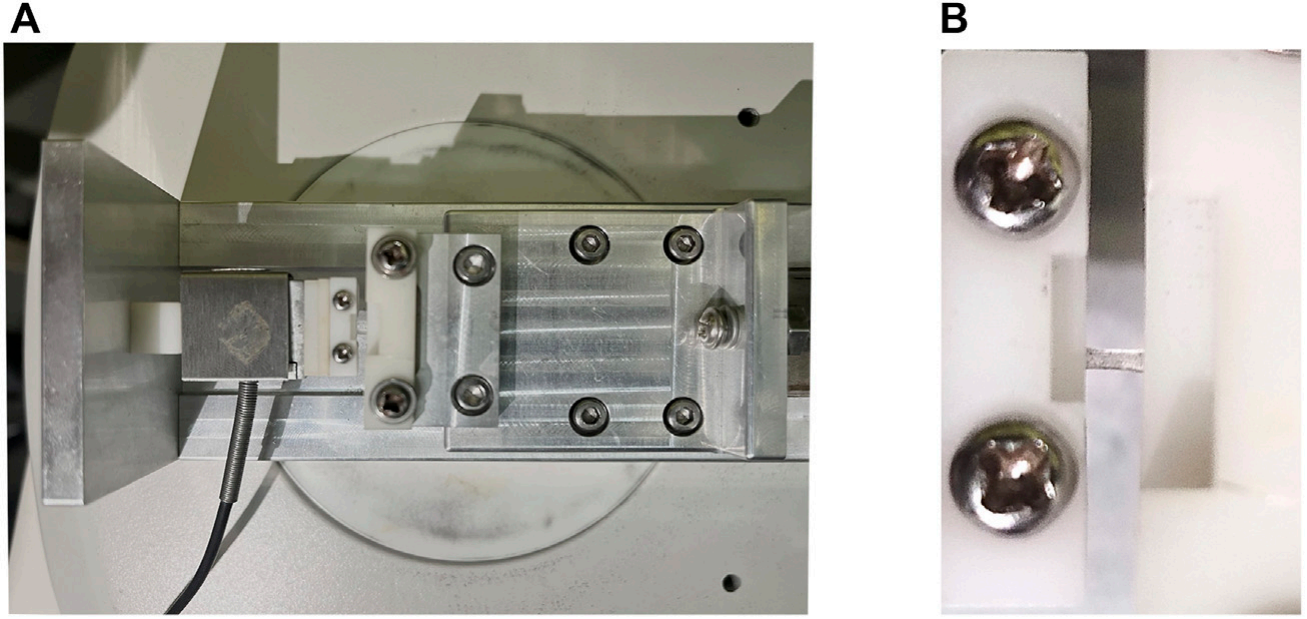
Photographs of the custom-built uniaxial tensile testing system. **(A)** The uniaxial tensile testing platform. **(B)** Corneal strip mounted between the clamps.

A custom-built tensile testing system (Figure 2) combined with customized SD-OCT was used in this study. The uniaxial tensile testing system includes a load cell capable of 1 N (ELFS-T3E-2L, Entran Devices Inc., Fairfield, NJ, United States) and a platform driven by a stepper motor. The SD-OCT imaging subsystem was similar to that used in previous corneal inflation experiments (Wang et al., 2018). It had a superluminescent diode (Part No. IPSDS804C, Inphenix Inc., Livermore, CA, United States), with a central wavelength of 840 nm, a bandwidth of 45 nm and an output power of 4.5 mW. Corneal cross-section imaging was obtained at an A-scan rate of 24 kHz. The axial and the lateral resolution of the OCT system were 8 and 21 μm, respectively. A CCD camera was incorporated to capture the shape of the sample. The entire system was controlled on a personal computer by a custom-designed program developed by LabVIEW (version 2009, National Instruments, Austin, TX, United States).

The corneal strip was mounted between the two clamps and moistened with phosphate buffered saline solution, after which the OCT probe was adjusted in alignment with the central area of the strip to record its structure during the testing process. The rate of clamp displacement was 0.05 mm/s and the maximal force was 0.25 N. Each specimen underwent two loading/unloading cycles for preconditioning.

### The Mathematical Analysis Procedure

**FIGURE 3:**
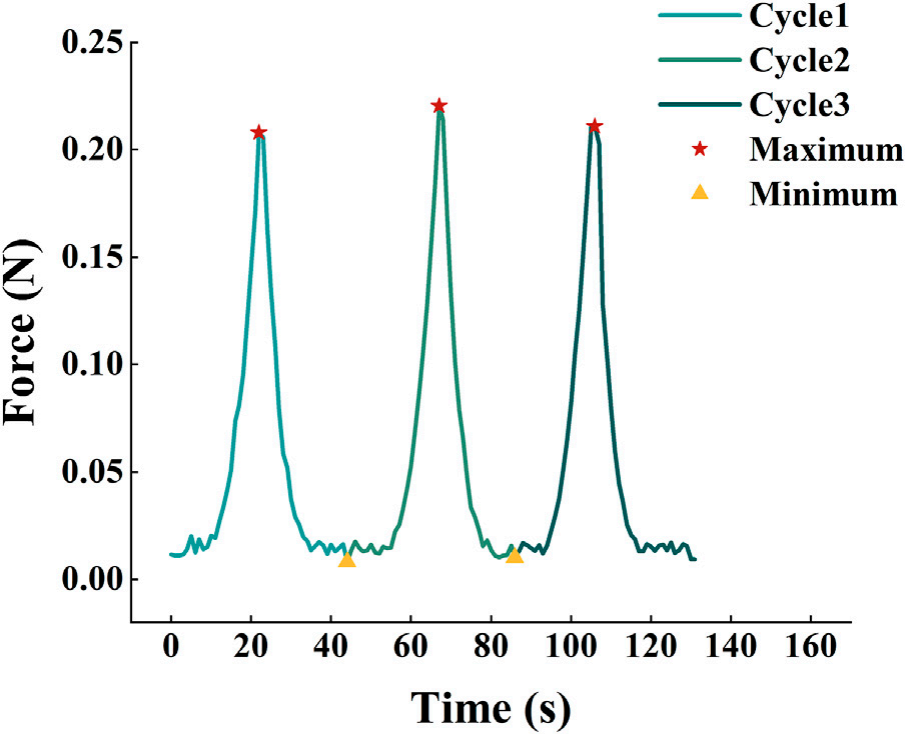
Division of three loading/unloading cycles. The force and the clamp displacement were divided into six segments by the five maximum and minimum force during the loading/unloading process.

The force *F* and clamp displacement *x* were divided into six segments by the five maximum and minimum forces during the loading/unloading process as shown in Figure 3. Each segment represents one stretching or slacking process. During one loading process, nonload section is located at the beginning of the curve caused by the initial bending of the corneal strips. The zero-load length (*l*_*0*_) was cut using the formulation (Liu et al., 2020)

$$\begin{array}{l} F\left(x\right)=d+\delta \left(x-l_{0}\right)\left[a\left(x-l_{0}\right)+b{\left(x-l_{0}\right)}^{2}\right]\\ \begin{array}{c} \mathrm{\delta =}\{\begin{array}{c} 0\mathrm{, x<}l_{0}\\ 1\mathrm{, x\geq}l_{0} \end{array} \end{array} \end{array}$$ (1)

where *d* is the average force in the zero-load length cause by placing the corneal strips.

The average-stress of the corneal strip can be calculated as:

$$\begin{array}{c} \mathrm{\sigma =}\frac{\mathrm{F-d}}{b\cdot h} \end{array}$$ (2)

where *b* and *h* are the width and thickness of corneal strips measured by OCT images, respectively. The strain of the strip can be calculated as:

$$\begin{array}{c} \varepsilon =\frac{x-l_{0}}{L_{0}+l_{0}} \end{array}$$ (3)

where $L_{0}$ is the original distance between two clamps.

**FIGURE 4:**
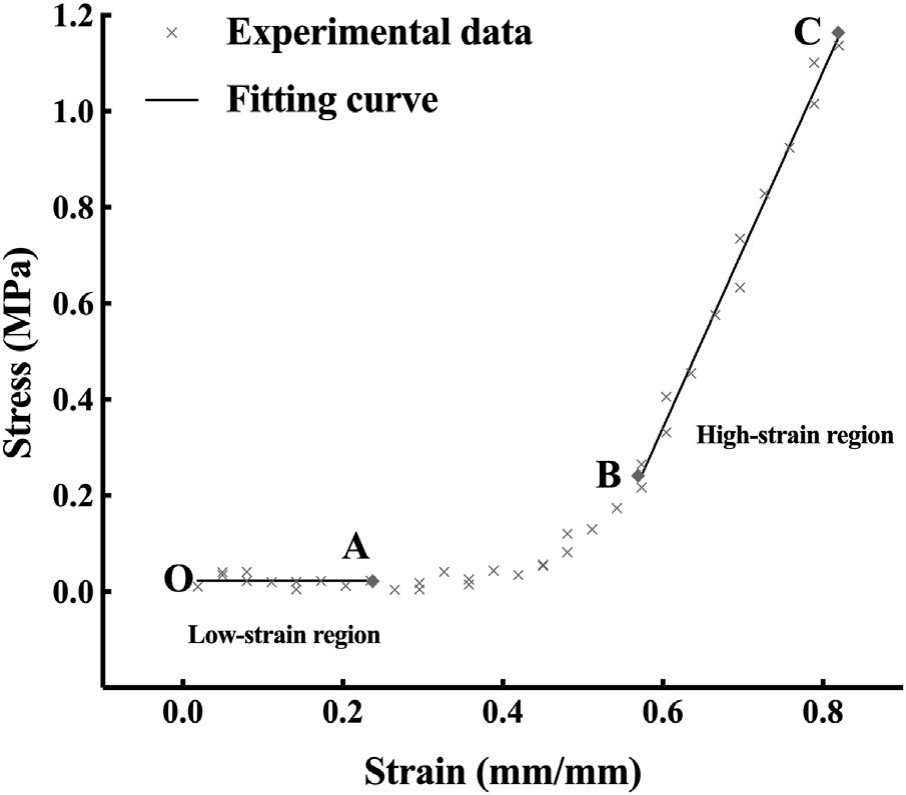
The stress-strain curve of a corneal strip.

According to the nonlinear property shown in Figure 4, the stress and strain curve was nonlinear and could be divided into three segments called toe region, heel region and linear region. The stress-strain relationship of the toe region and the linear region is linear like (Fratzl et al., 1998). Thus, the elastic property of corneal strips can be described by Young’s modulus, which is calculated as follows:

$$\begin{array}{c} E=\frac{\sigma }{\varepsilon } \end{array}$$ (4)

The Young’s modulus of the toe region and heel region are defined as low-strain tangent modulus (LSTM) and high-strain tangent modulus (HSTM), respectively.

### Statistical Analysis

All data were tested for normality using the *Shapiro-Wilk* test. The correlation of the elastic modulus with demographic and CorVis ST parameters were analyzed using *Spearman*’*s* correlation analysis, since the LSTM and HSTM were not distributed normally. All statistical analyses were performed using IBM SPSS software (version 22.0, IBM Corp., Armonk, NY). *p* value <0.05 was considered statistically significant.

## Results

A typical stress-strain curve is shown in Figure 4. With the growth of the strain, the stress increases slightly in the OA segment (the toe region), exponentially in the AB segment (the heel region) and linearly in the BC segment (the linear region). The mean LSTM was 0.204 ± 0.189 (range 0.010–0.641) MPa, and the mean HSTM 5.114 ± 1.958 (range 2.755–9.976) MPa.

**FIGURE 5:**
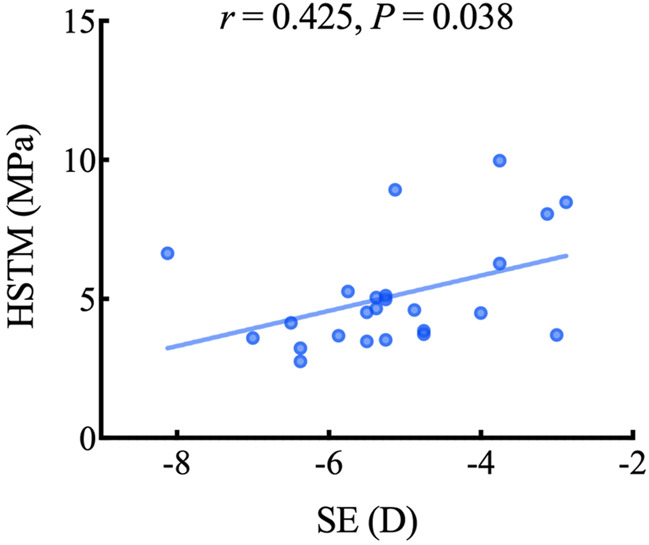
Scatterplot showing the relationship between the HSTM and SE. HSTM, high strain tangent modulus; SE, spherical equivalent.

Table 2 lists the correlation between LSTM, HSTM and the demographic and clinical characteristics. HSTM was positively correlated with spherical equivalent (SE) (*r* = 0.425, *p* = 0.038); that is, the higher the degree of myopia, the lower the HSTM, as highlighted in Figure 5. In addition, there was no correlation between HSTM and cylindrical diopter. Age, IOP, and central corneal thickness (CCT) were not associated with HSTM. No significant correlation was found between LSTM and demographic characteristics.

**TABLE 2:** Correlation between Young’s modulus and relevant clinical parameters (*n* = 24).

	LSTM		HSTM	
	r	*p* value	r	*p* value
**Age**	0.062	0.773	0.172	0.421
**Sphere**	0.117	0.587	0.395	0.056
**Cylinder**	0.175	0.412	0.175	0.412
**SE**	0.149	0.486	0.425	0.038^a^
**Km**	−0.262	0.216	−0.086	0.690
**CCT**	0.268	0.205	0.267	0.208
**IOP**	0.223	0.294	−0.049	0.819

LSTM, low strain tangent modulus; HSTM, high strain tangent modulus; SE, spherical equivalent; Km, mean keratometry; CCT, central corneal thickness; IOP, intraocular pressure.

a *p* < 0.05.

**FIGURE 6:**
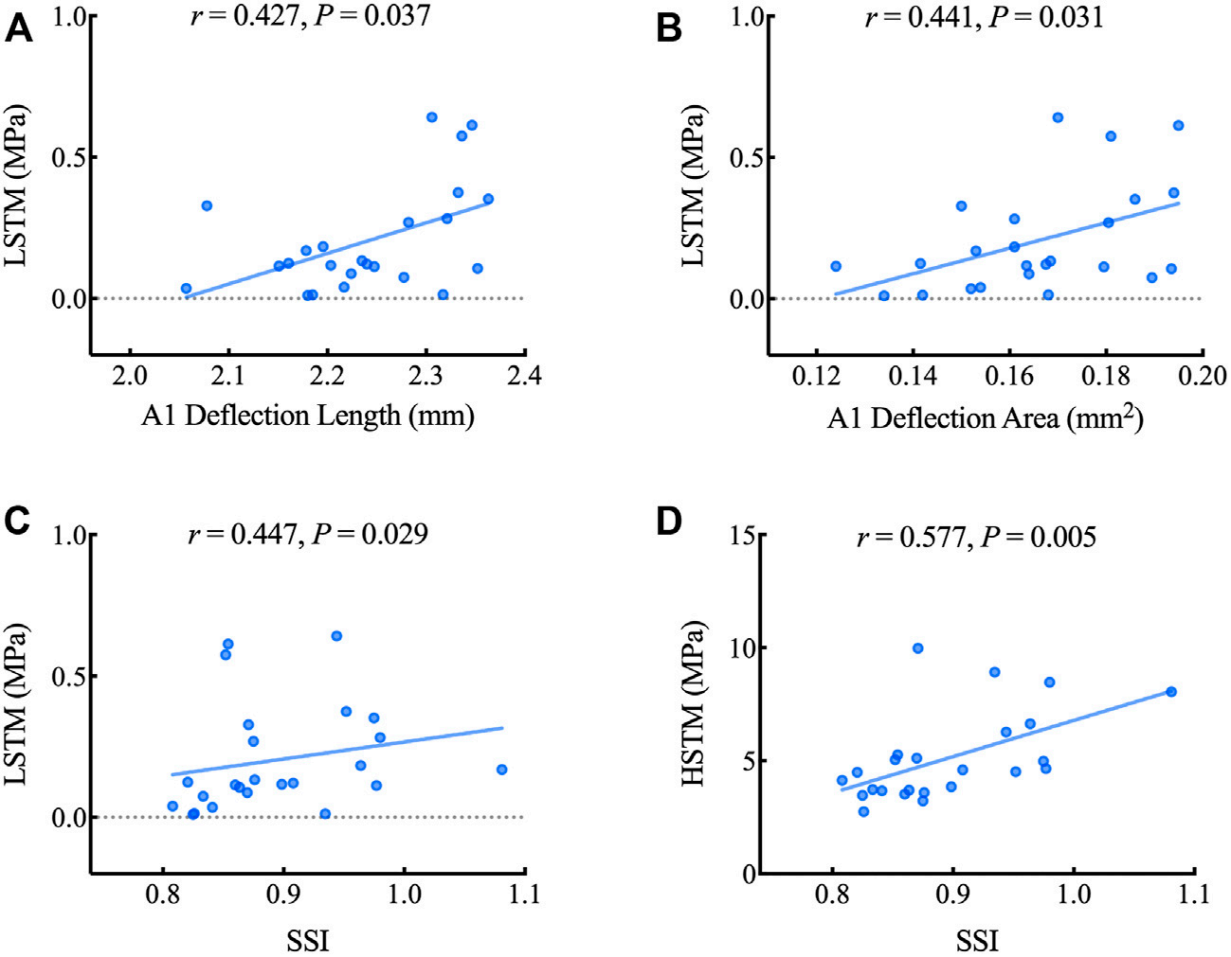
Scatterplots showing the relationship between the LSTM, HSTM and corneal dynamic response and stiffness parameters including A1 deflection length **(A)**, A1 deflection area **(B)** and SSI **(C, D)**. LSTM: low strain tangent modulus; HSTM: high strain tangent modulus; SSI: stress-strain index.

Table 3 illustrates the correlation between LSTM, HSTM and corneal dynamic response parameters as well as corneal stiffness parameters obtained by CorVis ST. As shown in Figure 6, LSTM was positively correlated with A1 deflection length (*r* = 0.427, *p* = 0.037), A1 deflection area (*r* = 0.441, *p* = 0.031) and SSI (*r* = 0.447, *p* = 0.029). HSTM was significantly correlated with SSI (*r* = 0.557, *p* = 0.005).

**TABLE 3:** Correlation between Young’s modulus and CorVis ST corneal dynamic response and stiffness parameters (*n* = 24).

	LSTM (MPa)		HSTM (MPa)	
	*r* (95% CI)	*p* value	*r* (95% CI)	*p* value
**A1 Deformation Amp. (mm)**	0.090 (−0.301-0.494)	0.676	−0.320 (−0.648–0.088)	0.128
**HC Deformation Amp. (mm)**	−0.313 (−0.645-0.071)	0.136	0.005 (−0.411–0.425)	0.982
**A2 Deformation Amp. (mm)**	−0.230 (-0.599–0.230)	0.279	−0.246 (−0.538–0.165)	0.246
**A1 Deflection Length (mm)**	0.427 (−0.017–0.734)	0.037^a^	0.001 (−0.445–0.447)	0.997
**HC Deflection Length (mm)**	−0.170 (−0.609–0.265)	0.426	0.206 (−0.251–0.578)	0.334
**A2 Deflection Length (mm)**	0.066 (−0.382–0.503)	0.759	−0.277 (−0.622–0.201)	0.189
**A1 Deflection Amp. (mm)**	0.373 (−0.057–0.697)	0.073	−0.266 (−0.643–0.132)	0.209
**HC Deflection Amp. (mm)**	−0.298 (0.635–0.109)	0.157	0.103 (−0.314–0.490)	0.632
**A2 Deflection Amp. (mm)**	0.236 (−0.206−0.576)	0.267	−0.018 (−0.473–0.459)	0.934
**Def. Amp. Max (mm)**	−0.313 (−0.645–0.071)	0.136	0.005 (−0.411–0.425)	0.982
**Deflection Amp. Max (mm)**	−0.251 (−0.619–0.149)	0.236	0.136 (−0.296–0.531)	0.526
**Deflection Amp. Max (ms)**	−0.112 (−0.548–0.397)	0.602	−0.239 (−0.575–0.189)	0.260
**A1 Time (ms)**	0.220 (−0.162–0.545)	0.302	−0.037 (−0.413–0.381)	0.865
**A1 Velocity (m/s)**	−0.220 (−0.573–0.174)	0.302	−0.027 (−0.514–0.419)	0.902
**A2 Time (ms)**	−0.319 (−0.659–0.032)	0.129	0.018 (−0.432–0.481)	0.933
**A2 Velocity (m/s)**	0.131 (−0.257–0.532)	0.543	−0.006 (−0.439–0.409)	0.977
**HC Time (ms)**	0.048 (−0.375–0.467)	0.824	0.017 (−0.343–0.346)	0.936
**A1 Deflection Area (mm** ^**2**^ **)**	0.441 (0.020–0.746)	0.031^a^	−0.052 (−0.493–0.373)	0.809
**HC Deflection Area (mm** ^**2**^ **)**	-0.296 (−0.645–0.113)	0.161	0.144 (−0.295–0.530)	0.501
**A2 Deflection Area (mm** ^**2**^ **)**	0.031 (−0.366–0.422)	0.885	0.039 (−0.403–0.504)	0.856
**A1 dArc Length (mm)**	-0.300 (−0.683–0.121)	0.154	0.197 (−0.276–0.568)	0.355
**HC dArc Length (mm)**	0.007 (−0.481–0.528)	0.974	−0.243 (−0.648–0.250)	0.253
**A2 dArc Length (mm)**	−0.010 (−0.428–0.420)	0.963	0.058 (−0.443–0.487)	0.788
**dArcLengthMax (mm)**	−0.070 (−0.500–0.408)	0.744	−0.087 (−0.510–0.338)	0.686
**WEM Max (mm)**	−0.194 (−0.536–0.269)	0.364	−0.212 (−0.552–0.213)	0.320
**WEM Max (ms)**	−0.363 (−0.736–0.085)	0.082	−0.117 (−0.485–0.280)	0.588
**Peak Dist. (mm)**	−0.163 (−0.566–0.240)	0.448	0.310 (−0.137–0.663)	0.140
**Radius (mm)**	0.224 (−0.139–0.571)	0.292	0.231 (−0.199–0.666)	0.277
**Max Inverse Radius (mm** ^**−1**^ **)**	−0.116 (−0.495–0.286)	0.589	−0.210 (−0.606–0.201)	0.324
**DA Ratio Max (2 mm)**	−0.286 (−0.591–0.131)	0.175	0.066 (-0.403–0.475)	0.759
**ARTh**	−0.183 (−0.552–0.199)	0.393	−0.227 (−0.601–0.190)	0.286
**Integrated Radius (mm** ^**−1**^ **)**	−0.337 (−0.676–0.082)	0.108	−0.130 (−0.587–0.323)	0.546
**SP A1**	0.068 (−0.318–0.458)	0.753	−0.150 (−0.561–0.323)	0.483
**SP HC**	0.243 (−0.136–0.595)	0.252	−0.135 (−0.515–0.311)	0.530
**SSI**	0.447 (0.027–0.745)	0.029^a^	0.577 (0.253–0.781)	0.005^b^

LSTM, low strain tangent modulus; HSTM, high strain tangent modulus.

a *p* < 0.05.

b *p* < 0.01.

## Discussion

Better characterization of corneal biomechanical properties helps in managing ocular diseases such as glaucoma (Susanna et al., 2019), ectatic corneal disease (Ambrósio et al., 2017) and in predicting surgical outcomes. The elastic modulus is the most frequently used description of corneal stiffness which reflects the capacity to resist an elastic deformation. The human corneal elastic modulus has been reported in several published works, despite some problems in the precise acquisition of sample thickness. This study aimed to propose a new method combining real-time imaging with uniaxial extension testing to obtain the corneal elastic modulus and investigate the correlation between the elastic modulus and *in vivo* biomechanical metrics.

The value of the human corneal elastic modulus obtained in our study was similar in magnitude as the results of Hoeltzel’s (Hoeltzel et al., 1992), which was 0.34 MPa. Owing to different experimental conditions including sample preservation, hydration, testing protocols, and calculation methods, the discrepancy of testing results between each study is unavoidable. For example, Xue et al.(Xue et al., 2018) reported that the LSTM of human corneal stroma was 1.17 ± 0.43 MPa in horizontal direction and 1.32 ± 0.50 MPa in vertical direction, and the HSTM was 43.59 ± 7.96 MPa and 51.26 ± 8.23 MPa, respectively. The difference in magnitude may be related to the difference in the testing machine and the experimental conditions. In their study, uniaxial tensile tests were performed using the IBTC-50 *in situ* tension and compression testing system, while in our study, a custom-built uniaxial testing system combined with SD-OCT was used. Furthermore, the OCT imaging subsystem utilized in our study is able to acquire the exact thickness of corneal samples, instead of estimating the sample thickness using surgical parameters. Regarding the experimental conditions, the corneal strip was bathed in normal saline during testing in Xue’s study, while in our study, the corneal strips were moistened with a drop of phosphate buffered saline solution prior to the test. In another relevant study published by Elsheikh and Alhassso (Elsheikh and Alhasso, 2009), 3-mm porcine corneal strips were tested as specimen, and the value of Young’s modulus fell between 0.343– 1.264 MPa. In addition, considering the heterogeneity in depth of corneal mechanical properties, our results may also be slightly different from those of other studies, as only corneal stroma was extracted and tested in our study, while other studies involved different layers.

This study introduced the concept of LSTM to describe the elastic modulus of the toe region which corresponds to the physiological level of stress. Relatively, HSTM, the elastic modulus of the linear region, reflects the mechanical behavior under stress that surpasses the physiological intraocular pressure (IOP). We found that subjects with a higher LSTM had a significantly larger A1 deflection length and A1 deflection area. These two parameters represent the length of the applanated segment of the corneal surface and the area of the applanated region at the first applanation, respectively. The applanation length is defined as the length of a line that describes the applanated segment of the corneal surface at the first applanation. Recent research (Roberts et al., 2017) has demonstrated that A1 deflection length/area is strongly affected by corneal stiffness. A stiffer cornea tends to have greater resistance to deformation, which generates a larger flattened length, and therefore area deformed. This observation is consistent with our results.

This study revealed the correlation between the *ex vivo* elastic modulus and SSI parameter provided by the CorVis ST machine, which is a newly introduced *in vivo* stiffness metric based on finite element modeling. Unlike most CorVis ST parameters, SSI is independent of IOP and corneal thickness, and has been validated through comparison with *ex vivo* experimental data of human corneas (Elsheikh et al., 2007; Elsheikh et al., 2010). This consistency could to some extent indicate the reliability of our data.

Interestingly, we also found that the HSTM decreased with the degree of myopia. The exact mechanism of the myopia remains unclear. Earlier work has confirmed that eyes with high myopia are biomechanically less stiff than those with low myopia by *in vivo* measurements (Plakitsi et al., 2011; Kang et al., 2018; Han et al., 2020). Our results are in agreement with previous findings, which may suggest the involvement of corneal biomechanics in the progression of myopia. A possible hypothesis might be that, given scleral thinning and localized ectasia of the posterior sclera in high myopia eyes (Rada et al., 2006), the cornea, as another load-bearing structure of the ocular wall, may have a similar alteration in mechanical strength. Further work is required to fully explore this potential mechanism.

Nevertheless, this study had some limitations. First, a relatively small sample size was unavoidable owing to the strict selection of experimental data. Conducting multiple comparison in correlation analysis further increase the possibility of false positive error in the results. Given that, a study with a larger sample size and multiple comparison error control is worthwhile to be conducted in the future. Second, we did not mark the direction of the corneal lenticule, although we chose samples whose astigmatism was <—0.5D to reduce the impact of asymmetrical corneal thickness distribution, and then minimize the effect of corneal anisotropy. Further studies can be followed to investigate whether the stiffness of cornea is directional in astigmatism cases. The elastic modulus measured in this study seemed to be variable, which may be due to the individual difference in mechanical properties and a relatively low sampling frequency in the tensile tests. Additionally, the viscoelastic behavior of cornea tissue was not fully considered when comparing *ex vivo* and *in vivo* measurements, while we focused on investigating the possible correlations, mainly in elastic properties.

In conclusion, we evaluated the elastic modulus of the corneal stroma under low strain and high strain using an advent uniaxial tensile tester incorporating SD-OCT. The LSTM was found to be accompanied by a larger A1 length and A1 deflection area measured by the CorVis ST machine, implying a relationship between these corneal dynamic response parameters and its intrinsic elasticity. The corneal elastic modulus seemed to be lower in highly myopic eyes, which may be a hint of corneal mechanical alteration. The attempt in our study may be a promising approach to better characterize corneal biomechanical properties and to verify the parameters provided by those widely used *in vivo* measurement machines for corneal biomechanics. This can further aid promoting procedures that mechanically interact with the cornea.

## Data Availability

The original contributions presented in the study are included in the article/Supplementary Material, further inquiries can be directed to the corresponding author.
